# Virtual and Augmented Reality in Post-stroke Rehabilitation: A Narrative Review

**DOI:** 10.7759/cureus.37559

**Published:** 2023-04-14

**Authors:** Rhutuja Khokale, Grace S. Mathew, Somi Ahmed, Sara Maheen, Moiz Fawad, Prabhudas Bandaru, Annu Zerin, Zahra Nazir, Imran Khawaja, Imtenan Sharif, Zain U Abdin, Anum Akbar

**Affiliations:** 1 Neurology, California Institute of Behavioral Neurosciences & Psychology LLC, Fairfield, USA; 2 Medicine, Medical University of Varna, Varna, BGR; 3 Intensive Care Unit, Sumeru City Hospital, Lalitpur, NPL; 4 General Medicine, Odessa National Medical University, Odessa, UKR; 5 Neurological Surgery, King Saud Medical City, Riyadh, SAU; 6 Medicine, Kakatiya Medical College, Warangal, IND; 7 Internal Medicine, All India Institute of Medical Sciences, Bhubaneswar, Bhubaneswar, IND; 8 Internal Medicine, Combined Military Hospital, Quetta, PAK; 9 Internal Medicine, Ayub Medical Institute, Abottabad, PAK; 10 Community Medicine, Quetta Institute of Medical Sciences, Quetta, PAK; 11 Medicine, District Head Quarter Hospital, Faisalabad, PAK; 12 Pediatrics, University of Nebraska Medical Center, Omaha, USA

**Keywords:** var, virtual reality (vr), augmented reality (ar), rehabilitation, stroke

## Abstract

Virtual reality (VR) and augmented reality (AR) are noble adjunctive technologies currently being studied for the neuro-rehabilitation of post-stroke patients, potentially enhancing conventional therapy. We explored the literature to find if VR/AR improves neuroplasticity in stroke rehabilitation for a better quality of life. This modality can lay the foundation for telerehabilitation services in remote areas. We analyzed four databases, namely Cochrane Library, PubMed, Google Scholar, and Science Direct, by searching the following keywords: ("Stroke Rehabilitation" [Majr]) AND ("Augmented Reality" [Majr]), Virtual Augmented Reality in Stroke Rehabilitation. All the available open articles were reviewed and outlined. The studies conclude that VR/AR can help in early rehabilitation and yield better results in post-stroke patients in adjunct to conventional therapy. However, due to the limited research on this subject, we cannot conclude that this information is absolute. Moreover, VR/AR was seldom customized according to the needs of stroke survivors, which would have given us the full extent of its application. Around the world, stroke survivors are being studied to verify the accessibility and practicality of these innovative technologies. Observations conclude that further exploration of the extent of the implementations and efficacy of VR and AR, combined with conventional rehabilitation, is fundamental.

## Introduction and background

Introduction 

Stroke, which was first referred to as "apoplexy" by William Cole in 1689, has become a significant cause of mortality and disability, particularly among the aging population worldwide [1-4]. It is defined as an acute onset of focal neurologic deficit caused by a cerebral blood circulation disorder that lasts for more than 24 hours or causes death with no other cause than of vascular origin and is also known as cerebrovascular accident (CVA) [5-7]. Ischemic and hemorrhagic are the two main types of strokes, with further classification based on subtypes. Ischemic strokes occur when blood flow to the brain is restricted due to a blockage in a blood vessel, while hemorrhagic strokes result from bleeding caused by a ruptured blood vessel in the brain filling into the intracranial cavity [7]. 

Modifiable and non-modifiable risk factors predispose individuals to stroke. Modifiable factors include hypertension, diabetes, obesity, or hypercholesterolemia, while non-modifiable factors include age, gender, sex, race, and family history of stroke [7-10]. Hemorrhagic strokes are less common than ischemic strokes, but they cause more severe outcomes, leading to death and disability-adjusted life-years (DALYs) lost [4,11]. 

A quarter of individuals over the age of 25 are predicted to be susceptible to stroke, and the incidence is higher in men than in women over the age of 65 [11-13]. However, age-adjusted rates for incidence, prevalence, mortality, and DALYs lost are expected to decline over time [14]. 

Stroke sequelae lead to significant disabilities [15]. It includes sudden weakness, numbness, or paralysis on either side of the body, difficulty speaking or understanding speech, and loss of vision in one or both eyes [4,16]. Motor impairments are also present in 80% of patients who suffer from stroke. Intravenous alteplase is the current treatment option for ischemic stroke, while the main goal of treatment for hemorrhagic stroke is to reduce intracranial pressure [17]. However, despite treatment, half of the patients with motor impairments experience a permanent disability that restricts their daily activities, social participation, and quality of life [18,19]. 

Rehabilitation is a crucial aspect of managing post-stroke complications, with the goal of improving the quality of life for patients [20]. Rehabilitation involves a multidisciplinary approach that focuses on enhancing and restoring natural bodily functions in various body parts. Unfortunately, research has shown that many stroke survivors face challenges regaining motor function, with only 50% of those with upper limb impairment regaining some motor function after six months. Similarly, only half of those with lower limb impairment can walk independently after rehabilitation [21]. Thus, there has been growing interest in exploring novel approaches, such as augmented reality (AR) and virtual reality (VR), as supplementary methods to enhance the rehabilitation process. VR entails using wearable screens, such as VR headsets, to immerse users in a completely synthetic three-dimensional (3D) environment, while AR involves overlaying digital information, like graphics or text, onto the user's view of the physical world using camera-enabled devices like smartphones or tablets [22,23]. Furthermore, mixed reality (MR) refers to a user environment created by merging the virtual and real worlds, allowing for real-time interactions between objects in both realms [24,25]. 

As opposed to traditional rehabilitation methods, AR and VR supplements offer several advantages, such as high repetition and intensity task specificity, objective feedback, greater user engagement, and improved motivation [25-27]. Repetition of movement is a crucial aspect of motor re-learning and facilitating neuroplasticity, which is essential for improving stroke patient rehabilitation outcomes [28-30]. 

In this narrative review, we will focus on the current understanding of the use of AR and VR in stroke patient rehabilitation, with a specific focus on the outcome of individualized AR/VR in rehabilitation following a stroke and their potential use in telerehabilitation for individuals residing in remote regions. 

## Review

Neural changes and disabilities after stroke

Stroke lead to an abrupt loss of neural function and damage to the central nervous system [31,32]. This creates an imbalance between the cerebral hemispheres, prompting cortical reorganization that provides a foundation for spontaneous recovery [33-35]. Cortical reorganization involves the transfer of functions from the affected brain area to the unaffected areas, followed by a shift back to the affected side after one to two weeks, with the perilesional area assuming control [36-38]. Neurotransmission recovery in the spared tissue further reflects the improvement in function [39-41]. Stroke can result in various disabilities, such as muscle weakness, altered muscle tone, numbness, incontinence, apraxia, aphasia, vision loss, and impaired activities of daily living [42-44]. Learned non-use may occur as a result of neural shock, causing progressive underuse of the affected extremity [45,46]. Hemiparesis, or one-sided weakness, is a common post-stroke symptom that contributes to activity restriction and reduced quality of life [47]. Upper limb impairment, referred to as upper extremity hemiparesis, can lead to limited function, with only a small percentage of patients regaining full function after six months [48-50]. Hemiparetic gait, characterized by spatiotemporal impairments, is also commonly observed after stroke, with many patients requiring physical support to walk [51-53]. Throughout the world, only 25% of stroke patients overall can return to their activities of daily living [54-56]. Social exclusion, depression, and decreased quality of life can also result from nonfluent aphasia and cognitive impairments after stroke [57,58]. Addressing both physical and psychological impairments is essential for effective rehabilitation, and VR/AR stroke rehabilitation and early activation changes in the sensory and motor systems may provide insights into restoring motor function [42]. 

Post-stroke rehabilitation

**Figure 1:**
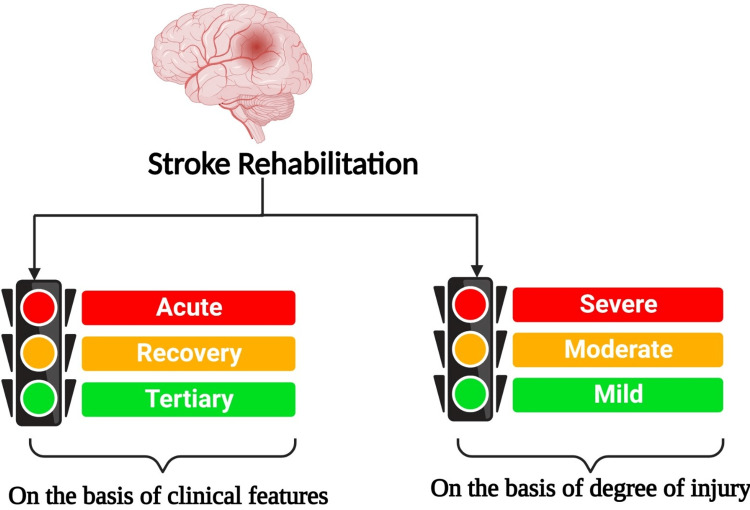
Classification of stroke rehabilitation Figure created with BioRender.com

Rehabilitation aims to promote neural plasticity by targeting abnormal connectivity within or between brain hemispheres [33,42]. It can be categorized based on clinical features and the degree of injury (Figure 1) [31]. 

Rehabilitation involves repetitive, challenging, motivating, and intensive exercises with meaningful and realistic tasks to enhance its efficacy [31,47]. Studies have shown that early rehabilitation after stroke can augment the duration of neuroplasticity, promoting cortical reorganization and compensatory mechanisms for improved function [59-61]. Interhemispheric facilitation has also been observed with bilateral extremities training programs [48,62]. However, traditional rehabilitation services are limited by cost, boredom, lack of motivation, economic burden, transportation difficulties, and inadequate medical facilities, which may lead to poor attendance and disappointment among patients [43,47]. To bridge this gap, there is a need for user-friendly neurorehabilitation solutions that can be accessed at home and specifically target activities of daily living for improved quality of life. 

VR and AR: subtypes and dynamics

**Figure 2:**
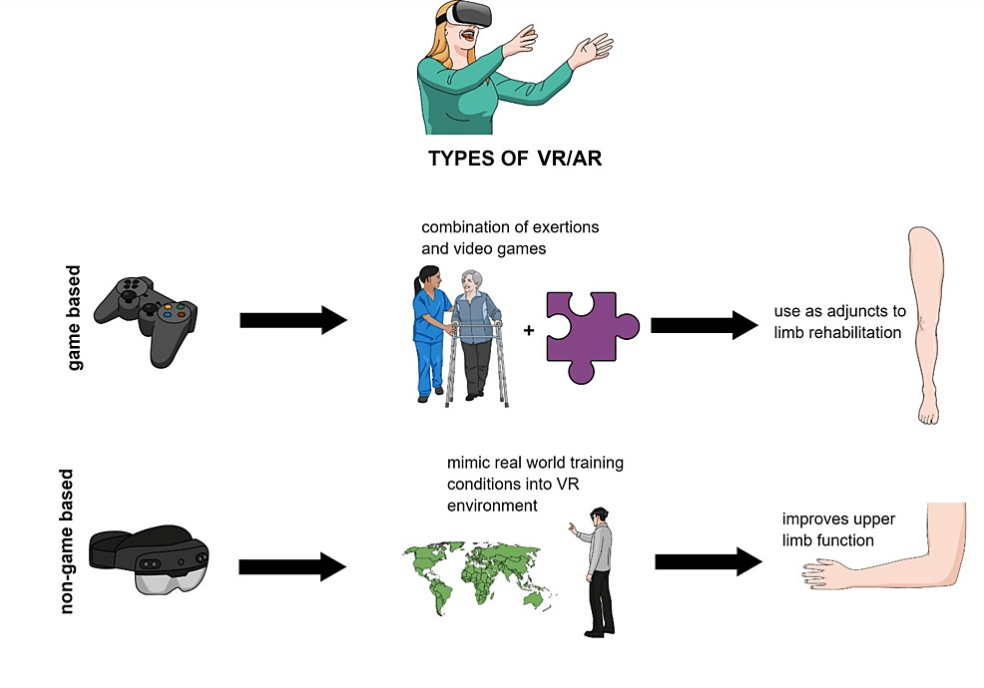
Subtypes of VR/AR VR: virtual reality; AR: augmented reality Figure created with Mindthegraph.com

VR/AR technology has emerged as a promising tool for neuro-rehabilitation. It can create an immersive virtual environment that mimics the real world and engages patients in task-oriented, repetitive, and intensive training [63-65]. AR technology can enhance VR by overlaying computer-generated images on real-world objects, creating a safer environment for patients to interact with natural objects [19,66]. Together, they form the VR/AR system, which can be categorized into game-based and nongame-based subtypes (Figure 2) [67-69]. VR/AR provides a means to guide and correct patients' motor behavior, enhancing their learning and motivation during rehabilitation [70-73]. 

VR/AR systems have been shown to yield better results when used as an adjunct to conventional rehabilitation [74-78]. For instance, the NeuroR system, based on mirror therapy, shows the user's image mirrored in a virtual environment, with a virtual arm replacing the affected arm [18,73,74]. The user attempts to physically perform arm movements using the impaired arm, and the electromyography (EMG)-based human-computer interface triggers the movement of the virtual arm. The virtual Nine-Hole Peg Test (9HPT) is another example of how conventional therapy techniques have evolved with the use of VR [18,75,79]. In this test, the user interacts only with the screen to perform the task. 

VR/AR also plays a role in aphasia therapy [76]. BTS-Nirvana (BTS Bioengineering, Milan, Italy) and EVA Park (EVA project, City, University of London, United Kingdom) are examples of VR/AR systems that allow users to work with task-based techniques [65]. EVA Park presents an ecological treatment wherein users can visit a world with shops, restaurants, and other services via avatars. BTS-Nirvana has virtual screens in which the user can interact with the tasks defined by the therapist through specific exercises [65]. These systems allow users to focus selectively on discrete cognitive abilities, including attention, executive functions, and memory. Overall, VR/AR technology has immense potential for neuro-rehabilitation, providing patients with an engaging and safe environment to improve their motor and cognitive abilities [77]. 

VR/AR techniques are showing promise as a way to improve outcomes in stroke rehabilitation. Conventional rehabilitation can become tedious, leading to decreased motivation and treatment adherence over time [19]. VR/AR offers a more engaging and personalized approach to rehabilitation, which may explain why it leads to significant improvements in motor, mental, and social functions [19,33]. 

Several studies have shown that VR/AR techniques can refine the motor control of stroke patients, filling the gap between the real and ideal world, and aiding in the early stages of recovery [19]. Motor-cognitive intervention concepts that target both physical and cognitive impairments are more beneficial for chronic stroke patients [47]. Moreover, VR/AR approaches are practical, feasible, automated, and can be conducted independently by the participants, thus releasing the workload on healthcare workers [80]. 

There are several examples of VR/AR systems that target specific aspects of stroke rehabilitation. For example, Gait-triggered mixed reality (GTMR) is a cognitive-motor dual-task with multisensory feedback targeted at lower-limb post-stroke rehabilitation. This platform has the inherent capability of adapting to the difficulty of a task to challenge the user continually but only making the task achievable [32]. Another example is Rehabilitation Gaming System for aphasia (RGSa), which provides lexical and syntactic training and has been shown to induce higher frequencies of language use and have lasting improvements [45]. Studies have been conducted on various VR/AR-based therapy as shown in Table 1.

**Table 1:** Studies on VR/AR-based therapy VR: virtual reality; AR: augmented reality; EEG: electroencephalogram; RAGT: robot-assisted gait training *Microsoft Corporation, Redmond, Washington, United States; **Motek Medical B.V., Houten, The Netherlands; ^Hocoma AG, Rockland, Massachusetts, United States; ^^ Rehabtronics, Edmonton, Canada

Reference	Therapy	Components	Mechanism	Impairments covered	Application	Benefits	Outcome
Leong et al. [19]	VR proprioceptive feedback training	VR headsets, Hand-held controller, Biofeedback sensor, Motion tracking system, Data analyzing software, Computer system to run VR software, and Appropriate rehab equipment.	Uses VR/AR techniques to improve motor control in stroke patients	Motor control	Early stages of stroke rehabilitation	Improves motor, mental, and social functions, fills the gap between real and ideal world, is practical and feasible, automated, and can be conducted independently by participants.	Use of VR in conjunction with a balance platform for proprioceptive training enhanced performance levels across various parameters, including low to high threat, eyes open or closed, and single or dual task scenarios.
Ko et al. [32]	Gait-triggered mixed reality (GTMR)	EEG monitor, Auditory/visual AR	Cognitive-motor dual-task with multisensory feedback tailored for lower-limb post-stroke rehabilitation	Lower-limb motor control, neuroplasticity	Stroke rehabilitation Spinal cord Injury	Cognitive-motor dual-task with multisensory feedback; immersive and motivational rehabilitation scenario that improves efficacy and accelerates motor recovery and neuroplasticity; provides behavioral and cortical information regarding rehabilitation progress	Gait speed, stride length, and balance, walking ability, balance, and quality of life in individuals with stroke were significantly improved in comparison to conventional therapy alone.
Kiper et al. [42]	Reinforced feedback in virtual environment (RVFE) therapy	Screen, VR headset, Motion tracking sensor, Biofeedback sensor	VR/AR system that demonstrates beneficial effect irrespective of the etiology of stroke	Motor impairments	Lower-limb post-stroke rehabilitation	Improves efficacy and accelerates motor recovery and neuroplasticity, adapts task difficulty, provides behavioral and cortical information regarding rehabilitation progress.	Implementation of augmented feedback by RFVE treatment with TR program is more effective than the same amount of conventional rehabilitation treatment to reduce upper limb dysfunction post-stroke.
Held et al. [56]	Augmented Reality for gait Impairments after Stroke (ARISE)	HoloLens 2* smart glasses, Sensor-based motion capture system	Provides an adjustable and personalized environment for gait and balance rehabilitation	Gait and balance impairments	Gait and balance rehabilitation	Improves gait and balance rehabilitation.	Gait adaptation during overground walking based on real-time feedback through visual and auditory augmentation.
Kayabinar et al. [44]	Robotic therapy augmented by VR/AR (RAGT)	Exoskeleton, Lokomat, C-Mill**, Armeo®^, ReJoyce^^	Uses VR to motivate users and increase their participation in rehabilitation	Motor impairments	Chronic stroke, spinal cord injury, Parkinson’,s multiple sclerosis, brain Injury, cerebral palsy	Increases participation in rehabilitation and motivates users.	Development in gait speed,dual-task performance, functional gains, and independence of chronic stroke patients with VR-augmented RAGT training, suggests the use of rehabilitation approaches in which VR is added, simultaneously, to functional training. Only RAGT approaches provided improvements in functional measurements, fear of falling, and independence levels. It was thought that this approach could contribute to patients’ rehabilitation process.
Alhirsan et al. [55]	Motorized treadmills with augmented feedback via VR/AR	Motorized treadmills, VR/AR System, Motion capture sensors, force feedback devices, biofeedback sensor, computer system, and data analyzer.	Enhance the body's intrinsic sensory feedback mechanisms	Motor impairments	Sensory feedback mechanism enhancement	Improves motor control.	Augmented feedback With VR interface and exergame tested positively in tasks involving motivation, attention, information, and attention.
Grechuta et al. [45]	Rehabilitation Gaming System aphasia (RGSa)	Computer/touch screen, Headphones/speaker, Camera	Lexical and syntactic training, and improves language use and construction of sentences	Aphasia	Post-stroke aphasia rehabilitation	Induces higher frequencies of language use and lasting improvements, improves anomia, conversation, elaborations, and comprehension of phrases, and construction of sentences -Self-paced for chronic stroke	Greater improvements in speech and communication abilities in comparison to those who received conventional therapy alone
Ko et al. [32]	Mobile brain/body imaging (MoBI)	EEG Headset, Motion tracking system, VR system, Computer system, Data analysis software, and Rehab equipment.	Exhibits neurological information to remote healthcare professionals, enhancing home-based rehabilitation	Neurological impairments	Remote/home-based stroke rehabilitation	Enhances home-based and allows for remote monitoring of progress.	Improvements in motor function, cognitive function, and quality of life in individuals with chronic stroke. The effectiveness of MoBI therapy depends on the severity of the stroke, the individual's motivation, and the duration of therapy

Despite the promise of VR/AR techniques, the lack of standardization in stroke rehabilitation leads to varied results in different systems. Additionally, some individuals using VR/AR reported limitations in mobility, and the head-mounted display caused some patients to experience nausea and headaches. Furthermore, VR/AR technology requires more experts to maintain and utilize the systems effectively. However, the advantages of VR/AR, such as better functional gains, incorporation of activities of daily living (ADLs) into VR/AR devices, individualized training, enhanced motivation, and enjoyment, make it an exciting area for further exploration in stroke rehabilitation [19,66,80]. 

It is important to acknowledge the limitations of our study. Firstly, our inclusion criteria only allowed for articles published in English, which may have introduced language bias. Secondly, we did not include unpublished or grey literature, which could have provided additional valuable insights to our review. Finally, the heterogeneity of the studies included in our review precluded a meta-analysis. Despite these limitations, our comprehensive review provides a useful overview of the potential of VR/AR in stroke rehabilitation, highlighting the need for further research in this area. 

## Conclusions

Our review highlights the promising role of VR/AR in enhancing neuroplasticity and improving the quality of life of stroke patients when used in conjunction with conventional therapy. Despite the limitations posed by targeted exercises and technical difficulties reported in some studies, the benefits of VR/AR, including its ability to engage patients in task-oriented, repetitive, and intensive training, are noteworthy. Our review also underscores the need for further research to explore the affordability and accessibility of VR technology in remote regions and clinics, as well as encourage neurologists to incorporate VR/AR as a tool for assessing stroke recovery.
